# Spatiotemporal relationship between ventricular expansion and flow propagation during early filling

**DOI:** 10.1186/1532-429X-11-S1-O91

**Published:** 2009-01-28

**Authors:** Thompson Richard, June Cheng Baron, Kelvin Chow, Jessica Scott, Ben Esch, Mark Haykowsky, Ian Paterson

**Affiliations:** 1 University of Alberta, Edmonton, AB Canada; 2 University of British Columbia, Vancouver, BC Canada

**Keywords:** Flow Propagation, Radial Expansion, Early Filling, Blood Flow Pattern, Relaxation Time Constant

## Introduction

Flow propagation refers to the delay in the onset of blood flow during early filling at more apical ventricular locations. The velocity of flow propagation (V_p_) into the left ventricle (LV) provides a preload insensitive estimate of LV relaxation, confirmed invasively by a strong negative correlation with the relaxation time constant (tau) [1, 2]. Clinically, V_p_ < 50 ms is commonly taken as evidence of abnormal diastolic function. No direct physical relationship between muscle relaxation and flow propagation has been previously illustrated. By simultaneous measurement of myocardial mechanics (radial expansion) and blood patterns throughout the LV and during early filling we expect to illustrate a correlated spatial and temporal relationship between the mechanics which drive blood flow and the resulting blood flow patterns.

## Methods

**Figure 1:**
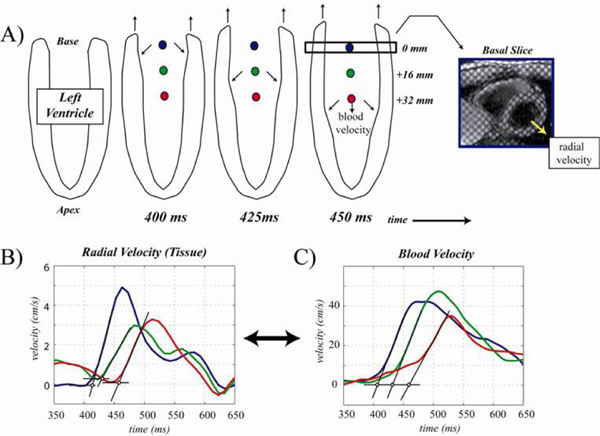
**Measuring deformation of the LV and blood velocity during early filling**.

Phase contrast and tissue tagged MRI were used to measure the timing of blood and myocardial tissue dynamics during early diastole (Siemens Sonata 1.5 T scanner). Experiments had a true temporal resolution of ~20 ms, interpolated to 10 ms for all analyses. The time of onset, t0 (time from QRS), of blood flow (phase contrast MRI) and radial expansion (tissue tagged MRI) were calculated at three 16 mm intervals from base to apex in 8 healthy volunteers. t0 was also evaluated as % systole (normalized to the time of aortic valve closure). Figure 1 shows an example of the measurement of t0 for both radial tissue motion (B) and blood flow (C). To account for residual low velocities that are unrelated to the early filling impulse (which can obscure the time of onset) the early diastolic blood and tissue velocities were linearly extrapolated to determine t0.

## Results

Propagation of flow and of radial expansion from the base to the apex (cartoon in Figure 1A) was observed in all subjects, as shown by the increasing delay in t_0_ as one moves towards the apex (see Table 1). The flow and radial expansion t_0_ values for each spatial position have similar means and good correlation (R in table). The resulting V_p_ values are also in general agreement.

**Table 1:** Table 1

	Base	+16 mm	+32 mm	Vp (cm/s)
Flow	384 ± 24/112 ± 7	411 ± 24/123 ± 5	436 ± 24/131 ± 7	66 ± 13
Radial Expansion	386 ± 31/114 ± 5	405 ± 32/121 ± 7	430 ± 32/129 ± 7	73 ± 10
R (t_0_)	.78	.73	.72	NS

t_0_ (ms/% systole)

## Conclusion

We demonstrated that spatiotemporal patterns of radial expansion (base to apex) are strongly correlated to conventional blood flow propagation during early filling, implying that muscle relaxation is related to V_p_ via the propagation of strain in the relaxing ventricle. These preliminary results show that MRI can be used to simultaneously quantify mechanics and hemodynamics, with good spatial and temporal registration (which is a strength of MRI as compared to ultrasound). It remains to be shown that the many other aspects of ventricular mechanical function (longitudinal strain, rotation, endo vs. epicardial motion, for example) are as closely coupled to hemodynamics as was shown in this study.
